# Integrated Health and Social Services for People With Chronic Mental Health Problems: People Are More Important Than Processes. Insights From a Multiple Case Study in Swedish Psychiatry

**DOI:** 10.3389/fpubh.2022.845201

**Published:** 2022-06-22

**Authors:** Karin Solberg Carlsson, Mats Brommels

**Affiliations:** Medical Management Centre, Department of Learning, Informatics, Management and Ethics, Karolinska Institute, Stockholm, Sweden

**Keywords:** integrated care, mental health, social services, informal care, case management

## Abstract

Three mental health organizations, one merged with, one formally cooperating with, and one without formal links to social services were analyzed through the experience of staff, patients and relatives in order to elucidate what approaches best promoted service coordination. Seventeen staff and eight patients or relatives, recruited from the three organizations, participated in semi-structured interviews, guided by pre-selected categories derived from previous research about coordination and care processes. Directed content analysis was used to identify and categorize meaning units. Both staff and patients raised the same concerns. Organized collaboration between psychiatric care and social services addressed only some of patients' challenges. More important was patient access to financial and social assistance. The organizational arrangements were not referred to, whereas case management was seen as crucial. In many instances relatives have to act as case managers. Service integration in mental health has to include, in addition to social services, other authorities like social insurance and employment agencies. A case manager knowledgeable about all welfare services is best positioned to promote that “extended integration”. Relatives often have to take this responsibility to support this fragile group of patients. This observed importance of case management is supported by previous research in mental health and primary care. The role of relatives should be acknowledged and supported by those services.

## Introduction

The suffering and costs of mental health problems are a concern all over the world. As regards treatment, the evidence shows widespread under-coordination of the many health and social services which people with mental health problems usually need (1) and also sub-optimal involvement of patients and informal carers (2).

Advances have been made in clinical treatments, but possibly less progress has been achieved in better coordinating the help which different professionals, community services, and patients' friends and family (“informal carers”) can provide. Many different interventions to improve coordination have been described and some tested (3). Models of care combining a number of components or characteristics have also been tested, such as collaborative care models, primary care behavioral health integrated care models and some patient centered home models for mental health patients (3–5).

There is limited empirical research on how to enable patients and informal carers to take a greater role, as desired and appropriate, in their recovery and in self-care. Actions to promote and support self- and “co-care” could be added to or emphasized to improved coordination but would require different coordination arrangements so as to be more responsive to and enabling of patients and informal carers. It is possible that such improvements in coordination could yield as much return on investment for reducing suffering and costs as improvements in medications (6).

Our previous research reported two mental health collaborations with social services in a Swedish county with care coordination characteristics that were different to those of the traditional less integrated approach and which we will term here “mental health configurations” (2, 7, 8). The collaborations that we previously reported implemented two distinct strategies of service coordination for more than a decade. One is an example of structural integration of psychiatric and social services, whereas the other is a close cooperation between separate organizations that showed a “functional integration.” Our longstanding partnership research with those two units provided us with access to data, and to staff, patients and relatives to examine their perceptions of coordination. In the study reported below, we included a third psychiatry unit in from the area which did not involve formally-structured relationships with social services so as to allow comparison with a different model and increase the generalisability of the study to other health systems.

One cause of under-coordination is thought to be the structural separation of providers. At the macro level, hospitals and medical specialists are organized, and often financed and regulated in a different way to primary care professionals, as also are social services. Public social care services are governed and financed separately and subject to different regulations, for example, concerning clinical information storage and access.

Different methods are used to improve care coordination, and some have been effective for coordinating care for mental health patients (9). However, there are significant challenges in implementing these methods and models and a lack of research into which implementation strategies and methods might be effective for enabling coordination changes of different types (10–12).

There are few studies that have systematically investigated the reported experience of clinical staff, patients and relatives about health and social service integration in Sweden or elsewhere. These perceptions can contribute to understanding examples of effective coordination, types of under-coordination perceived to be significant, possible causes of under-coordination, and real examples to use in training for improvements.

The purpose of our research was to study the experience of persons with chronic mental health problems and their relatives, as well as those of professional staff regarding different forms of integrated health and social services. The aim also was to identify successful strategies to achieve effective coordination of services, as experienced by patients and relatives.

## Materials and Methods

### Study Design

The design used qualitative research methods to collect data chosen as relevant from previous research about coordination and care processes. Data collection was through semi-structured interviews with patients experiencing chronic psychosis and their and informal or “close-carers,” and with professional staff in three types of mental health service configurations in Sweden. Data was analyzed using directed content analysis.

### Study Setting

In Sweden, self-governing counties are responsible for funding, providing and commissioning healthcare. Municipalities organize social services, as well as care for the elderly and people with chronic physical or mental disabilities. Consequently, healthcare and social services, equally important to people with health problems, are provided by two different public authorities.

The first configuration included in the study was a combined county-owned hospital department of psychiatry and municipal social services agency. The second was a county-owned hospital-based department of psychiatry in another part of the region, with a close informal collaboration with the municipal social services agency there, and co-location of services. The third is a department of psychiatry with no formal collaboration with external actors. The settings thus represent a variety of different types of organizations that provide care for patients with chronic psychosis.

### Study Participants

Twenty-one health professionals (managers, physicians, nurses and assistant nurses) were contacted through email, telephone, or both, and eighteen chose to participate. As regards health professionals, three interviews were carried out with two health professionals jointly and the others were individual interviews. Patients and relatives were recruited using a poster requesting involvement at each facility which generated two patient interviews. Six further interviews were carried out by recruiting those who responded to health professionals asking their patients if they were willing to participate. These six interviews involved: one interview with a patient accompanied by a relative, one only a relative, and the remaining interviews with patients alone. The patients' ages ranged from twenty-six to sixty, with between 1–10 years since receiving their diagnosis of psychosis. Initially we retrieved the participants' contact information from key informants at each of the three facilities and as the interview process progressed, the interviewees provided us with additional contacts. In all eighteen women and eleven men were interviewed. A total of twenty-five interviews were performed, either individually or in pairs, at the three mental health care facilities in the county (Detailed list is available upon request).

The semi-structured interview form is exhibited as Appendix I.

### Data Collection

Most interviews lasted 1 hour on average (ranging from 20 min to1 h and 30 min). Semi-structured interview guides were developed for interviews with managers, health care professionals and patients/relatives. The guides were based on the assessment of previous research noted above in order to probe the areas of interest and ensure some comparability in the interviewees' answers. The guides elicited interviewee perceptions about the facilities' strategies for integrating care from different providers, such as health care, home aid, financial aid, social services, as well as other care provided by the municipality, and the patients' and relatives' perceptions of the care received.

Each interview followed a standard procedure. We informed the participants about the purpose of the study, that participation was voluntary, that they could withdraw from the study at any time, that all data would be handled confidentially, and that maximum effort would be made to maintain anonymity when presenting the data. Each participant gave their oral informed consent for recording the interview twice: once before the interview started and once after the recording started, and these were subsequently transcribed *verbatim*.

### Analysis

Each transcript was first read to create familiarity with the full material before analyzing the data in detail. Based on the questions in the interview guides, we created a codebook with pre-selected categories in NVivo 12 (www.qsrinternational.com/nvivo-qualitative-data-analysis-software/), thus using directed content analysis to identify and categorize manifest and latent meaning units. We also coded other comments made by interviewees about coordination that were not immediately related to any interview inductively under a separate code (“Other”). Latent meaning units were identified and analyzed by noting moods, such as laughter and tone of voice, during the interviews and in the transcripts. If we had doubts regarding the tone in which something was said, we listened to the audio recording. If we were uncertain about which code to use for the latent message, we looked at the larger context in which something was said, such as reading what was said before leading up to the latent meaning unit of interest.

To ease data processing and improve transparency, we condensed and translated the coded meaning units, and sorted them in framework matrices in NVivo 12. The themes derived from the categorized findings are presented in the findings section below together with quotations. These were translated by a native English speaker to ensure that the meanings of the quotations were as close to the Swedish verbatim as possible.

### Ethical Considerations

The study protocol was submitted to the Stockholm Regional Research Ethics Board, which approved the study (DNr 2017/104-31).

## Results

The results of this study are presented in two parts. First, in order to shed light on how the three studied organizations implemented the goal to provide integrated services the views of staff and managers on successful as well as poor coordination are presented. Second, the experience of patients and relatives are reported including what had worked well and had been unsatisfactory.

### Part I: Staff Experience

The staff described factors that facilitated the coordination of care, as well as the problems. We report those described commonly for all three of the configuration models we studied, as well as those specific to one or two of the integrated, coordinated or traditional configuration cases.

### Case Coordination Meetings and Care Planning Are Difficult to Arrange, Coordinate, and Follow Up in an Efficient Manner

Since 2009, for all patients “Coordinated Individual Plans” (in Swedish: “*Samordnad Individuell Plan*”) have to be designed, usually at meetings were all providers involved are present. Those “CIP meetings” were said to work especially well when many participated, including representatives of the state Employment Agency and Social Insurance Agency. However, the CIP meetings were described as an unpractical way of meeting since they are difficult to arrange physically and administratively. It was reported that persons with the right mandate to make decisions are often not present, and agreements made during CIP meetings are not always followed and goes unnoticed for a long time, putting the patient at risk. Sometimes, informal discussions separate to patient care take place.

### Communication and Recording Procedures Often Lack Standardization and Traceability

Concise and clear reporting between those involved was said to facilitate care coordination, in all of the models we considered be it face-to-face, over telephone, or through written notes. A problem common to all was that reporting becomes untraceable when not documented in an EPR. Non-standardized ways of communication were said to slow down processes and make information sharing untraceable since each actor has its own documentation system, which in turn requires double documentation. There is for example no system or routine for handling shared ICP documents. Non-standardized communication was said to lead to negative incidents such as missed primary care appointments and missed information between different health care specialties. Participants expressed wishes to either have one documentation system for all actors, or access to those of their collaborators.

### Standardized Care Pathways Could Improve Quality of Care

Aligned and standardized care pathways were suggested by some as a solution for improving quality of care, patient safety, care continuity, sustainable collaboration between actors, and ultimately equitable care. The county standardized care processes were said to support and structure daily work and facilitate the training of new employees at two of the facilities. However, the fully integrated facility does not have access to the county care process maps and have developed its own. Process maps are available to all staff at all three facilities.

### A Comprehensive Systems View Is Beneficial for Collaboration but Can Risk Blurring the Agreed Professional Boundaries

The staff said that broad knowledge of the health system in general is needed for a good collaboration between actors. Shared leadership, where organizations benefit from each other's knowledge, and case manager roles that act as a connection center between actors for patients were mentioned as ways to achieve this. The speedy integrated services have reduced the number of in-patient days and facilitate fast action during acute alarms and early onset of disease (before diagnosis). However, there were several examples of where a professional's broad knowledge led to overstepping their formal boundaries, such as nurses performing house cleaning duties for the psychiatric social services or acting as mediator between two external actors, and a psychiatric out-patient care facility setting up a somatic care facility for psychosis patients to catch somatic illnesses early and check patients urine for drugs, since the rigid opening hours for this service at the substance abuse center do not fit the capabilities of psychosis patients, and their failure to follow through can lead to withdrawal of social welfare aid. In addition, case managers were said to help the patients fill out forms to apply for social allowance and support the patients to apply for the municipal social services, since all patients who wish to get such help need to apply to the municipality.

“(Collaboration) *can be interpreted in different ways, where some interpret it as “everybody can do everything,” while the more correct way is to interpret it as professional conversations to help the patient in the best possible way, thus completing each other professionally, not smudging boundaries by sharing every task”* [interview # 25].

“*The municipalities do not always have the right competencies to care for our type of patients, and when the municipalities do not take enough responsibility when needed, the county council staff overstep their boundaries for the sake of their patients”* (10).

“*When a patient has assisted living services in the home, those personnel are the ones who have the responsibility to accompany the patient to doctors' visits, dentist and such, and sometimes we have to motivate both the patient AND the personnel to go there”* (13).

“*Patients have to see far more people than they can handle and sometimes I have to coordinate between two external actors* (16).

### Patients' and Relatives' Involvement Is Helpful but Can Be Hindered by Individual Psychosis Patients' Attitudes Toward Giving Consent to Information Sharing

Patients' and relatives' active involvement in care planning was described as helpful in decision making and setting goals throughout the care process, with the patient being an active part of the care team rather than being “in the center with the team around them.” However, the need for patients' consent to sharing information between actors can hinder collaboration and was described as unfortunate. In some cases the CIP document was developed to formally allow the psychiatric care and social services staff to break secrecy and exchange information with each other about the patient.

### Misalignment of Actors Complicates, and Sometimes Hinders, Collaboration

A misaligned system of actors complicates collaboration across organizations, and several factors were mentioned: financial instability due to different funding mechanisms in the different organizations jeopardize collaborative work, mismatch between the actors' responsibilities and the patients' actual needs, and misalignment of roles, responsibilities and mandates between collaborating actors that delay aid to patients. In addition, there is no existing formalized definition of and/or standard for collaboration, although national authorities require it by law. There is a need for consensus among the different actors. Often, a new collaboration between actors has to be set up for each new patient. Participants also mentioned that a mismatch in reimbursement mechanisms can lead to misalignment in actors' focus, thus hindering fully integrated work processes.

“*The Employment Office would not help my patient get a job because he was on sick-leave from his old job, which he will never be able to return to, because they would only help him if Social Insurance Agency had referred him there, and Social Insurance Agency will not refer him because he has a steady job, even though he is on sick-leave from it”* (16).

“*Since there are many young people with psychosis, it is unfortunate for them that the Social Insurance Agency only helps people with an income, and our young patients are left outside, getting no money and very little help from the Employment Office as well, and they end up in social welfare from the municipality which would not happen if they had become ill five years later, and it would be better if the unemployment office and the Social Insurance Agency would be in the national guidelines, which is unfortunate that they are not now”* (15).

### Top Management Collaboration

Weakened collaboration between organizations' top management was said to weaken the once strong collaboration throughout the lower levels of the organizations partnering voluntarily. Concerns were raised that the focus on how they collectively deliver care to patients will be jeopardized and they stressed that ensuring quality on an organizational level should be viewed as a form of “specialization”. However, it was also stressed that a narrow focus on collaborative work processes may lead to inequitable care for those patients whose needs do not require this type of collaboration.

### Small Size of Geographical Catchment Area Can Be Beneficial to Improving Collaboration

Having a small collaborative community, either like the structurally integrated organization or the co-located facility, was said to facilitate information sharing and direct contact between actors. If not co-located, field trips and shared lectures with actors with whom one shares patients with were mentioned as successful ways to familiarize oneself with the actors involved. Co-location of actors was also said to facilitate speedy information sharing and gaining a broader knowledge of the system. The non-integrated facility expressed difficulties in collaborating with too many municipalities and their “care neighbors” in their geographic catchment area due to the differing routines for collaboration in each municipality. It was said that matching geographical catchment areas for all key actors could facilitate collaboration. Several interviewees said that the success or failure of the collaboration depends to a large extent on the person you are collaborating with on the other end. The role of standardized equitable care was expressed by a patient:

“*I am reluctant to move to another city, in case care is different somewhere else”* (19)

### Proximity of Services Is Often Helpful but Is Not Always the Sole Factor That Guides Patients' Choice of Facility

The nature of the psychosis disease result in patients often seeking help in their geographical catchment area rather than traveling further, which is also in line with the clinics' wishes since they are required to provide timely care in the home in acute situations, which cannot be guaranteed if the patient lives too far away. It should, however, be mentioned that one relative interviewed had a different view and expressed that being able to choose where to receive rehabilitation was helpful since they were then able to pick a rehabilitation center close to work rather than home, which enabled the whole family to attend group activities. The changing demography of the psychosis patient group has influenced the psychiatric care facilities to adjust the services they provide to better fit the patient needs.

“*We meet the patients where they are in their work life more often than having them come here and break isolation.”* (1)

### Part II: The Views of Patients and Relatives

The patients and relatives expressed how they perceived the components and processes of the care and services they received, as well as actions they have taken to achieve a decent life situation.

### Staff Is Attentive to Patient and Family Members' Involvement

Patients said they appreciate that health care listens to them and understands their situation and that the old hierarchies in health care are gone. Furthermore, health care's welcoming of family members' active involvement in care planning was described as positive and patients from all three facilities described their respective facilities as being well-coordinated internally. However, some patients had experienced that psychiatric care has the wrong treatment focus for them, i.e. minimizing symptoms rather than curing their cause, that their examination and treatment “toolbox” is often too narrow, and that more preventive care is needed. One interviewee said it feels like mental illness is a “second class illness and receives second-hand health care”, and that patients with these problems are not taken seriously by the National Board of Health or society at large. Patients also wished for a speedier process of receiving a correct diagnosis.

### All Social Services Need to Be Included

Good collaboration between health care and the social services was described as helpful when applying for financial aid. However, patients experienced problems on a system level regarding regulations in social services and external actors not being aligned with each other, or with patients' needs. It is difficult to understand the rules of the Social Insurance Agency, the Employment Office and the municipality, and the rigid rules put on the actors often hinder organizations from collaborating on solving a patient's problem, such as helping a patient on sick leave from a job which he/she will not be able to return to, to switch jobs.

“*Our son can perform certain work tasks and I think society would benefit from that, but the authorities such as the unemployment office and Social Insurance Agency do not support him in getting such a position, and instead they have to deal with me calling them pretty often to take his fight”* (21)

In addition, one family member had experienced extreme difficulties in receiving timely and adequate services from social service actors when the son/daughter was residing in an apartment in a neighboring municipality but had the mailing address at the parent's house so that they could help out with administration.

“*…there is no humanity in those organizations and we have seen no signs of a will to collaborate in the organizations outside the county council […] they show no respect for my son”* (21)

### Patients' Capacity to Participate in Care Planning Differs Between Individuals

The patients' role and knowledge about their care plan differs in the three facilities. Patients in one of the facilities had never partaken in planning their own care together with health care professionals whereas patients the other two facilities partake in the development of their care plan and are aware of its details, revision routines and location. However, when the responsibility of coordinating and administrating tasks related to care and social services is put on patients, it is often mismatched with their capabilities, and often goes unnoticed by the professionals involved. An example of such a situation is when a patient's formal sick-leave period was coming to an end and he/she was asked to apply for extended sick leave, but was too sick to even fill out the necessary paperwork and find out what type of sick-leave his/her diagnosis was eligible for.

“*The more ill you are, the more you have to know yourself to get in contact with the correct type of care”* (18).

“*It is very easy to believe that our daughter is doing much better than she actually is because she is capable of acting that way for short periods of time, and she may have fooled a few doctors and nurses”* (24).

Focus needs to be put on patients' needs, and responsibilities need to be matched with the patient's level of capacity, be it higher or lower than initially perceived.

“*NN is in need of minor support to remind him of paying bills and care for his home, but someone suggested he would get a trustee, which made us very sad because that would mean that basically many of his rights would be taken away from him and he is not that incapable of doing things”* (21).

### Relatives Often Need to Act as Coordinators of Services

The relatives interviewed said that family members often take the responsibility placed on the patient to coordinate and execute the tasks put on the patient, and they often have to make sure that decisions between actors in the health system are properly documented, such as the patient's consent to information-sharing across organizations and to family members, which sometimes is overlooked. However, the nature of the disease sometimes cuts connections to the patient's family members, thus creating inequalities in patients' ability to handle complicated health related events. Participating relatives further explained that they would not have been able to support their loved ones, had their work hours not been as flexible as they are, had they not taken vacation days, had they not been on sick leave for some time, had they not had a strong marriage, or recently retired. In addition, the relatives interviewed said that they did not receive enough, if any, information about what was wrong with their family members in the acute phase. One relative said that it took a very long time, over a month, for the family before they got an appointment to speak to the physician in charge of their hospitalized daughter and another said that when their son was hospitalized they were not allowed to find out where he was for two or three days.

### A Case Manager Can Act as a “Spider in the Web”

Patients and relatives from all three organizations described that well-coordinated care was facilitated by having a professional contact person who takes the full responsibility for coordinating the care and external services that the patients need. They expressed a wish for a case manager with a helicopter view of the health system who can coordinate the patients' appointments and services. These roles were formalized as case managers in two of the facilities, whereas the role was “spontaneously” taken by a social worker for one of the interviewed patients in the remaining facility. Patients with case managers often described the case manager's role as being a “spider in the web” or an “attorney” who testifies to external and internal actors that what the patients say is true, and work in favor of the patient to solve problems.

“*…the case manager can accompany us to appointments outside health care to sort of testify that… I am not another hysterical mother”* (21)

The case manager's role having a broad spectrum of capabilities and taking on large amount of responsibilities was described as helpful when forwarding patient information to the physician, reminding the patients about appointments, keep track of financial situation, help write letters, CVs, job applications and other applications, and accompanying the patient to appointments to, primary care, the Social Insurance Agency and the Employment Office. The fragmentation of services creates a risk that actors lose track of patients, that patients lose energy, and often have to repeat their medical history to all actors involved. However, one patient also expressed that having a case manager as a first contact person sometimes makes it difficult to get direct contact with their physician when medical adjustments are needed and wished for direct contact with the physician through a digital platform.

### Co-location of Services

Co-location of two or more actors was said to be practical for patients needing multiple services.

Patients and a relative (of another patient) gave examples of what actions they have taken themselves to achieve a decent life situation. These were: reaching out to services in other municipalities, using a relative's address in a municipality with better care or services, giving relatives user ID information so that they can help pay bills and do administration, or simply staying where they live now due to the fear of not receiving good care elsewhere, although the desire to move closer to family and friends remains.

“*I get less paid now that I am on sick-leave and I am glad that I am able to rent out a room in my apartment, and that I do not eat very much”* (22).

## Discussion

To our knowledge this is the first study on integrated services for persons with severe chronic mental health conditions that is based on in-depth interviews with both patients, relatives and mental health staff. This multiple case study design took advantage of the existence of three different organizational forms of service integration in one Swedish county: one provider organization that had merged county-owned health services (including specialized mental health care) with municipal social services, one long-standing voluntary, but organized collaboration between psychiatry and social services that are co-located, and a department of psychiatry that had no formal links to social services. Those organizations are examples of structural, functional and no formal integration.

The findings suggest that, from the perspective of those interviewed, formal organized collaboration between psychiatric care and social services address only some of patients' challenges. As, or more, important was patient access to services for financial and social assistance, including social support, and coordination with these services. These included government agencies such as the Social Insurance Agency (granting, i.a., sick leave compensation), the Employment Agency and Social Welfare. Patients have to contact these agencies on their own and are most often dependent on the support of others to do this, usually a relative.

Psychiatry departments cooperate with primary care and social services that often are organized by a number of specialized units and sub-contractors. The greater the number of potential collaborators, the more important is the formal case manager role. Our interviewees reported that this task is best carried out by a person who has broad knowledge of the local and regional system and a comprehensive inter-professional network. Many noted that this takes years of experience to develop, and if that person leaves, it takes time for their replacement to build the same level of help and rapport with patient and their relatives.

Co-location of psychiatric and social services was said to be important for collaboration and makes coordination of services smoother and more rapid than otherwise. A drawback observed was that co-location led to informal activities and task-sharing, running the risk of blurring professional boundaries and, as a consequence, deterioration in professional competence.

Neither patients nor staff made any reference to organizational arrangements between psychiatry and social services when they reflected on service integration and coordination. In all the three configurations we studied, a case manager was consistently reported to be the most important service integrator. Familiarity with all relevant agencies and personal relations to actors involved will promote successful coordination.

One of our findings was that relatives in many instances had to perform the functions of a case manager. This was also found in a systematic review that highlighted the importance of the role of relatives especially during the first symptoms of a psychosis (13). Similarly, this was reported by Del Vecchio et al. (14) who also found that parents are more important than siblings or spouses as informal case managers. Taking this role may require a relative to reduce working hours in order not risk one's health and avoid sick leave (15). This was also reported by some of our relative interviewees. Doebler et al. (16) report on the association between time spent by a relative on case management and the risk of mental ill-health. One finding then is that the input of relatives should not be taken for granted, and that they need support to ensure that they are not exploited or overburdened (17). This also raises the need to organize extra support for patients who have no such relatives or friends to help.

Our observation of the limited importance of formal organizational arrangements is echoed by a rapid review on to what extent service integration contributes to the physical health needs of people with severe mental illness (18). The authors of that review could not report research that showed integrated care arrangements to be more effective than “ordinary care.” Although a number of models of integrated mental health services were identified, many of those were considered by this study to be poorly described and did not show effective ways of providing well-coordinated services to persons with severe mental health problems.

However, many different meanings of integrated care make it difficult to compare different studies. In the 1980s and 1990s many multi-professional community mental health teams were formed (19). Those teams offered “integrated treatment,” meaning that many team members rather than its single members were involved in therapy and other treatments. One variant named “assertive community treatment (ACT) teams” were later found by randomized trials to improve clinical outcome and adherence to treatment in patients with schizophrenia (20). A version of ACT for patients with psychosis is “intensive case management” that restricts the number of patients to 20 and provides those with “high-intensity input.” Although the literature provides only limited evidence, there are indications that such an intervention reduces hospitalisations and improves social functioning (21).

Woody et al. (22) performed a systematic review of what made multidisciplinary teams responsible for persons with severe mental illness successful. Communicating with respect among team members and across disciplines, well defined team leadership and including patients and relatives were related to positive outcomes. Unclear purpose and lack of agreed ways of working made teams ineffective. The importance of dedicated team leadership was also emphasized by a study of 135 multidisciplinary mental health teams in the English National Health Service (23).

An Irish study analyzed a multi-disciplinary mental health team that included social workers also. It raised the concern that professional role blurring could arise, calling for negotiations on division of work to maintain professionals' practice identities (24). This is in line with the observations made in one of our cases.

Teams are also important to support case management in psychiatry. Care coordination benefits from respectful team member interactions and good communication as well as from agreed structures and processes (25). This study is one of the few on case management in mental health. Considerably more reports can be found from primary care. As an example, a Canadian study reported that nurse case managers involved patients with chronic conditions in developing and executing their individualized services plans, which patients and relatives felt led to improved communication, coordination, shared decision-making and improved transitions between service providers (26).

Given the importance of case management with good continuity, one of the principal findings of our study, and considering the challenges of work-force shortages and high staff turnover, it is worth contemplating whether recent advances in digital health could be utilized in improving service integration for mental health patients. Iorfino et al. (27) studied in a systems dynamics model potential advantages of “technologically-enabled care coordination” in mental health. In that scenario, online technology was employed to facilitate the delivery of multidisciplinary team-based care. Teams were supposed to design individual treatment and care plans collectively. In a simulation exercise this scenario was predicted to reduce self-harm hospitalization, suicide deaths and emergency department visits, whereas merely increasing mental health service capacity or applying standard telehealth (offering existing services online) had a lower impact. Employing e-health applications has been shown to enhance patient-provider communication and care planning in collaboration, which can be expected to improve the integration of care services. Such positive results have been achieved by implementing a web-based “individual care plan” shared by patients and providers (28) and a web-portal offering a toolbox for provider-guided patient self-management and a peer-support forum (29).

We conclude that providing integrated services to persons with severe mental health disorders is of great importance. It goes beyond “integrated treatment,” although multi-disciplinary teams may positively contribute to seamless services for this patient group. Our study shows that integration has to include, in addition to psychiatric and social services, other important public authorities like social insurance and employment agencies. To reach that “extended integration” is best guaranteed by experienced case managers with good knowledge of all welfare services and a vast inter-professional network. However, in many instances relatives have to act as case manager. Mental health services have to acknowledge the input made by relatives, give them support and involve them in an extended support network for persons with severe mental ill-health. Organizational forms of integration and collaboration, such us structural, functional or non-formal integration seem to be of less importance.

This qualitative study has several strengths but also limitations. One advantage is that the study had access to three different forms of organized cooperation between psychiatry and social services. It adds credibility to our conclusion that the organizational form is less important than case management and extended support networks. We were able to include both staff, patients and relatives in our study, and in groups of sufficient size to show saturation. The fact that all stakeholders groups had similar views strengthens the trustworthiness of findings reported. A limitation is that the study covers a short period of time for patients having a chronic disorder, and that the sample includes only patients and relatives with recent contacts with mental health services, and that those were volunteers, running a risk of introducing bias into the study.

The study was performed in three psychiatry units in one Swedish county. We do not claim that the results are applicable to other contexts or organizational settings, although it is plausible that the importance of case management for seamless services would be valid elsewhere also, given other reports referred to in this study.

## Data Availability Statement

The raw data supporting the conclusions of this article will be made available by the authors, without undue reservation.

## Ethics Statement

The studies involving human participants were reviewed and approved by Stockholm Regional Research Ethics Board, approval DNr 2017/104-31. The patients/participants provided their written informed consent to participate in this study.

## Author Contributions

KC and MB conceptualized and planned the study, analyzed the coded data, and drafted the manuscript. KC performed the interviews and the primary coding of the interview data. Both authors contributed to the article and approved the submitted version.

## Funding

The study was funded by the Swedish Research Council (Grant No. 2014–2710).

## Conflict of Interest

The authors declare that the research was conducted in the absence of any commercial or financial relationships that could be construed as a potential conflict of interest.

## Publisher's Note

All claims expressed in this article are solely those of the authors and do not necessarily represent those of their affiliated organizations, or those of the publisher, the editors and the reviewers. Any product that may be evaluated in this article, or claim that may be made by its manufacturer, is not guaranteed or endorsed by the publisher.
