# Supplementary material for: Integrated Health and Social Services for People With Chronic Mental Health Problems: People Are More Important Than Processes. Insights From a Multiple Case Study in Swedish Psychiatry
Source: Front Public Health. 2022 Jun 22;10:845201. doi: 10.3389/fpubh.2022.845201 (PMC9257072; doi:10.3389/fpubh.2022.845201)
Supplement: Supplementary file 1 [file Table_1.DOCX]

Appendix 1: Semi-structured interview format

**For patients and close-carers**

**How old are you?**

**How long ago was your first contact with psychiatry?**

What was the profession of the first person have that you came into contact with?

What happened when you received your diagnosis. How long did it take?

**During this past year, which professionals have been in contact with you and been involved in your care?**

(for example: Psychiatrist, primary care physician, nurse, nurse assistant, psychologist, therapist, dietician, occupational therapist, physiotherapist, social worker, home care, pharmacist, other)?

Do you know which one of these to turn to when you need help?

Do you know what to expect when you contact them?

Do you get the help you need fast enough?

Before this past year, were you in contact with any of the above mentioned? Which ones?

**Have you received the care you need during the past year?**

What has worked well? What has not worked well?

What advice has health care given you to manage your health? (“Early signs” of illness phase)

Between your visits, do you feel that you have enough information to manage your health?

**Is there a contact person for the care and services you need? Do you have a *main* contact person?**

What is his/her profession?

What role/function does this person play in your care? Is it working?

Does this person refer you to the correct care and service contacts you need?

Does this person make these contacts for you?

Is your main contact person aware of your health and mental state and uour needs?

Does your main contact person seem to be updated on the care and services you receive?

Does your main contact person help you get the care you need from other clinics?

Is your main contact in contact with you even when you receive care elsewhere?

**With whom do you and your main contact person collaborate?**

How do you experience the actors’ collaboration?

Have they given you varying information or told you different things?

Do they seem to know who performs which tasks in your care? Do you know?

Do they make sure that these tasks are performed?

Do you have to organise the services you need yourself?

Have the persons responsible for your care always had the information needed for your care close at hand?

**Altogether, how well organised would you say your care is?**

Does communication between the actors you have described worked well?

Have you had to repeat information that should be in your EHR? Why?

Which changes do you think are necessary to be made regarding how actors communicate or collaborate? (Suggestions)

**Do you know if a care plan has been made for you in the past year?**

Have you seen it or taken part in developing it?

Did any of your relatives partake in the planning of your care?

If not, would you have wanted them to?

In practice, is the plan in writing?

Where is it kept?

**For clinical professionals**

**Could you please tell us about your clinical qualifications and how long you have worked at this facility?**

**How does your organisation currently work with psychosis patients? Its strengths? Problems?**

**How do you coordinate the care for psychosis patients?**

Does every patient have one or more professionals acting specifically as coordinators? (If no, ask interviewee to give a thorough description of how care is coordinated and move to question 5)

**Tell us about the coordinator’s role.**

To what extent does this person have knowledge about the patient’s needs and wishes? (If not, who does?)

To what extent is this person aware of other care and service actors involved in the patient’s care? (If not, who is?)

To what extent is this person aware of the patient’s current health and mental state? (If not, who is?)

Holistic perspective – to what extent does this person track the patient’s care contacts? (If not, who does?)

**Tell us about the division roles/responsibilities in your organization and in external actors’ organisations.**

How do different care and service providers communicate among each other?

Can you give examples of when patients have received conflicting information from different care and service providers?

Can you give examples of how different care and service providers have assessed patients’ needs and functions differently among each other?

Can you give examples of successful coordination between actors?

Can you give examples of lacking coordination between actors?

Have you experienced that a person you work/collaborate with has not been aware of which actions have been decided upon/taken?

How do you experience collaboration with primary care physicians?

**How has evidence based medicine and care planning been used within the past 12 months?**

How many patients receive an individual care plan?

Who is responsible for following up that the decisions made in the care plan are followed through?

Have you ever developed such a care plan? How is it done? Is it a written document?

Are the patient’s own treatment goals brought up and documented? To what extent are patients and relatives part of developing the care plan?

Is coordination or collaboration documented in a specific way? Is it available for you when you need it?

How important do you perceive the patient’s coordinated care plan to be? How important is inter-professional teamwork?

When a shared planning meeting is needed, do all relevant actors partake?

**Describe the care pathways for psychosis patients.**

To what extent are the standardised care processes for psychiatric patients used by your organisation?

Is the coordinated/collaborative work documented in a standardised way in any EHR system?

**Do you always have complete patient information from psychiatry *and* social psychiatry at hand when you need it?**

Do you know of any incident when a patient has been at risk because you have not had access to necessary or important information from psychiatric care and social services?

To what extent does this collated information help you make informed decisions?

Is the information you need always available?

Do you have access to the systems containing this information?

Is information documented in a timely manner?

Can you always contact other co-workers to discuss or receive important information about a patient?

**What improvements in communication, information sharing and collaboration do you see as necessary? How can this be done?**

**Are there any other person you think we should talk to regarding the issues we have discussed today?**

Staff?

Patients?

Documents?

**For managers**

(Managers at psychiatric care and service units within the Stockholm County Council)

Background

How long you have worked at this facility and what is your professional background?

Service history

Has your facility experienced any major events of importance for us to know in the past few years (2015-2017)?

Surroundings and context:

What in your organisation’s context has facilitated your improvement work until present day?

What in your organisation’s context has hindered your improvement work until present day?

Content, goals and purpose:

What actions were taken?

What was the purpose of this action?

Process and implementation:

How were these actions implemented? (specific method, specific projects)

Which actors specifically were involved?

Results:

Which results were expected?

Which results have you observed?

How many patients does your organization see?

How many of them are psychosis patients?

How many are in-patients?/out-patients?

Within management there is talk about customers. Who would you say are your customers?

*Summarise and name the different customer segments.*

Which customer segments do you consider to be the most important?

What do you see as the main difference between your facility and a traditional psychiatric care facility regarding patient groups and customers?

Customer segment Patient: Why do they choose to come to your facility and not elsewhere? (How do your patients find out about your facility?)

How has your (coordinated) way of working affected:

Your patients?

Their relatives?

Your staff?

Health outcomes? (for example readmissions, suicide attempts)

How do patients find their way here? (Long-term? Short-term? Referrals?)

Are there specific customer segments/patient groups who currently do not seek your help but who you would prefer to see? How could this be solved?

Are there patient groups you would prefer to ”avoid”? How could this be solved?

What are your most important resources used to handle the different customer segments you have mentioned? [name one segment at a time]

With whom (actors) do you collaborate to uphold your organization or facility?

Are there other actors you would like to set up collaboration with? Why?

What are your main costs? (fixed? Flexible?)

Has the way you coordinate your work affected your organization financially?

What are your sources of revenue?

How does the current reimbursement system/mechanisms affect how you organise and deliver care? (give examples)

Are there any customer segments that are prioritized or down-prioritized due to the current reimbursement mechanisms? (give examples)

*Summarise the business model components*

What do you consider to be the most important thing(s) to improve?

What facilitates collaborative work? What makes it more difficult? (Internal and external factors)

SLSO has implemented standardized this past year.

How far has your organization come in implementing the psychosis care processes?

How do you work with its implementation?

Concluding questions

Is there anything more you would like to mention?

Are there more persons we should talk to or documents that you can share with us that could complement the information we have received regarding your organization?

Thank you for your time. We will contact you with a summary of the results.
